# Mortality from cutaneous melanoma: evidence for contrasting trends between populations

**DOI:** 10.1054/bjoc.1999.1243

**Published:** 2000-06

**Authors:** G Severi, G G Giles, C Robertson, P Boyle, P Autier

**Affiliations:** Division of Epidemiology and Biostatistics, European Institute of Oncology, via Ripamonti 435, Milan, 20141, Italy; Cancer Epidemiology Centre, Anti-Cancer Council of Victoria, Melbourne 3053, Australia

**Keywords:** melanoma, mortality, trends, prevention programmes, sun exposure

## Abstract

In recent years several reports have been published concerning trends in melanoma mortality in different countries, some of which have indicated that rates are beginning to fall. Many of these reports, however, have been based on small populations and have used different forms of statistical analysis. Our objective was to analyse systematically to what degree the epidemic of melanoma mortality had evolved similarly in different populations and whether there were any divergent trends that might increase our understanding. Instead of using all available data, we focused on countries with a minimum time series of 30 years and a minimum of 100 deaths annually in at least one sex from melanoma. We first inspected sex-specific age-standardized mortality rates and then performed age-period-cohort modelling. We found that the increase in mortality observed after 1950 was more pronounced in the age group 60–79. Statistical modelling showed a general increase in mortality rates in generations born after the turn of the century. Downturns in mortality, essentially in women and starting with generations born just before World War II, were found in Australia (where the earliest decreases were noted), the Nordic countries and the USA. Small decreases in rates in more recent generations were found in the UK and Canada. However, in France, Italy and Czechoslovakia, mortality rates were seen to be still increasing in recent cohorts. Our analysis suggests that populations are at different places on the melanoma mortality epidemic curve. The three trend patterns we observed are in agreement with time differences between populations with respect to the promotion of sun protection and the surveillance of pigmented skin lesions. © 2000 Cancer Research Campaign


*British Journal of Cancer* (2000) 82(**11**), 1887–1891
© 2000 Cancer Research Campaign
DOI: 10.1054/ bjoc.2000.1243, available online at http://www.idealibrary.com on IDEAL®

### Mortality from cutaneous melanoma: evidence for contrasting trends between populations

**G Severi[1], GG Giles[2], C Robertson[1], P Boyle[1] and P Autier[1]**

[1]Division of Epidemiology and Biostatistics, European Institute of Oncology, via Ripamonti 435, Milan 20141, Italy; [2]Cancer Epidemiology Centre, Anti-Cancer Council of Victoria, Melbourne 3053, Australia

**Summary** In recent years several reports have been published concerning trends in melanoma mortality in different countries, some of which have indicated that rates are beginning to fall. Many of these reports, however, have been based on small populations and have used different forms of statistical analysis. Our objective was to analyse systematically to what degree the epidemic of melanoma mortality had evolved similarly in different populations and whether there were any divergent trends that might increase our understanding. Instead of using all available data, we focused on countries with a minimum time series of 30 years and a minimum of 100 deaths annually in at least one sex from melanoma. We first inspected sex-specific age-standardized mortality rates and then performed age-period-cohort modelling. We found that the increase in mortality observed after 1950 was more pronounced in the age group 60–79. Statistical modelling showed a general increase in mortality rates in generations born after the turn of the century. Downturns in mortality, essentially in women and starting with generations born just before World War II, were found in Australia (where the earliest decreases were noted), the Nordic countries and the USA. Small decreases in rates in more recent generations were found in the UK and Canada. However, in France, Italy and Czechoslovakia, mortality rates were seen to be still increasing in recent cohorts. Our analysis suggests that populations are at different places on the melanoma mortality epidemic curve. The three trend patterns we observed are in agreement with time differences between populations with respect to the promotion of sun protection and the surveillance of pigmented skin lesions. © 2000 Cancer Research Campaign

**Keywords**: melanoma; mortality; trends; prevention programmes; sun exposure

Since World War II, the number of deaths due to cutaneous malignant melanoma (CMM) has increased sharply in most fair-skinned populations (Armstrong and Kricker, 1994; Swerdlow, 1990). However, the rate of increase has varied by country (La Vecchia et al, 1999). For example, up to 1990, the increase in CMM mortality was found to be 30–50% greater in Southern and Central Europe than in Northern Europe (Swerdlow, 1990; Balzi et al, 1997).

There is also evidence that CMM mortality rates are plateauing or beginning to fall in recent generations in the USA and in Australia (Scotto et al, 1991; Rousch et al, 1992; Giles et al, 1996). These declines in mortality are, however, restricted to recent birth cohorts while death rates continue to increase in older generations. In European countries, in 1990, there was no indication of a similar phenomenon (Balzi et al, 1997), except in data from Scotland (1979–1994) which showed a stabilization of mortality in women (all ages) and in men younger than 65 years old (MacKie et al, 1997).

Because of the apparently contrasting trends in CMM mortality in different countries, we decided to investigate whether the decreases recently observed in Australia and the USA were emerging elsewhere. Our strategy was to limit our analysis to fair-skinned populations that possessed long time series and large annual numbers of CMM deaths. In this way, we attempted to base our models on the best data available rather than including every population irrespective of data quality and chance variation.

*Received 18 October 1999*
*Revised 17 January 2000*
*Accepted 17 January 2000*

*Correspondence to:* G Severi

### MATERIALS AND METHODS

CMM mortality data were obtained from the World Health Organization Cancer Mortality Data Bank. Populations were selected for analysis that were predominantly Caucasian (except Japan) and that were of a sufficient size to provide adequate numbers of events (an average of 100 CMM deaths annually in at least one sex) over a period of at least 30 years. Germany was excluded because of missing data in some years for the former eastern part of the country. The Netherlands was not selected because of insufficient numbers of deaths. Because of small numbers, the Nordic countries of Denmark, Finland, Sweden and Norway were combined. In combining the Scandinavian countries we did not take into account a priori evidence concerning trends in these populations. Altogether nine countries or macro-regions had adequate data available from at least 1960 to 1994 (1992 for Italy, 1991 for Czechoslovakia and 1993 for the USA and the Nordic countries). The populations we selected are representative of most of the world regions affected by CMM.

Annual data were obtained for each sex by 5-year age groups. Rates were age-adjusted to the World Standard Population for the age groups 30–59 and 60–79. The average annual percentage increase in rates between 1960 and 1990 was computed for the two age groups in order to explore the general trends in CMM mortality in young and old people.

As previously reported (Swerdlow, 1990), there was a pronounced peak in rates in France in 1968 probably due to misclassification problems, so for this year French data were interpolated from adjacent periods. In the USA, the inclusion of African-Americans in the denominators and numerators is likely to have diminished substantially the magnitude of the rates

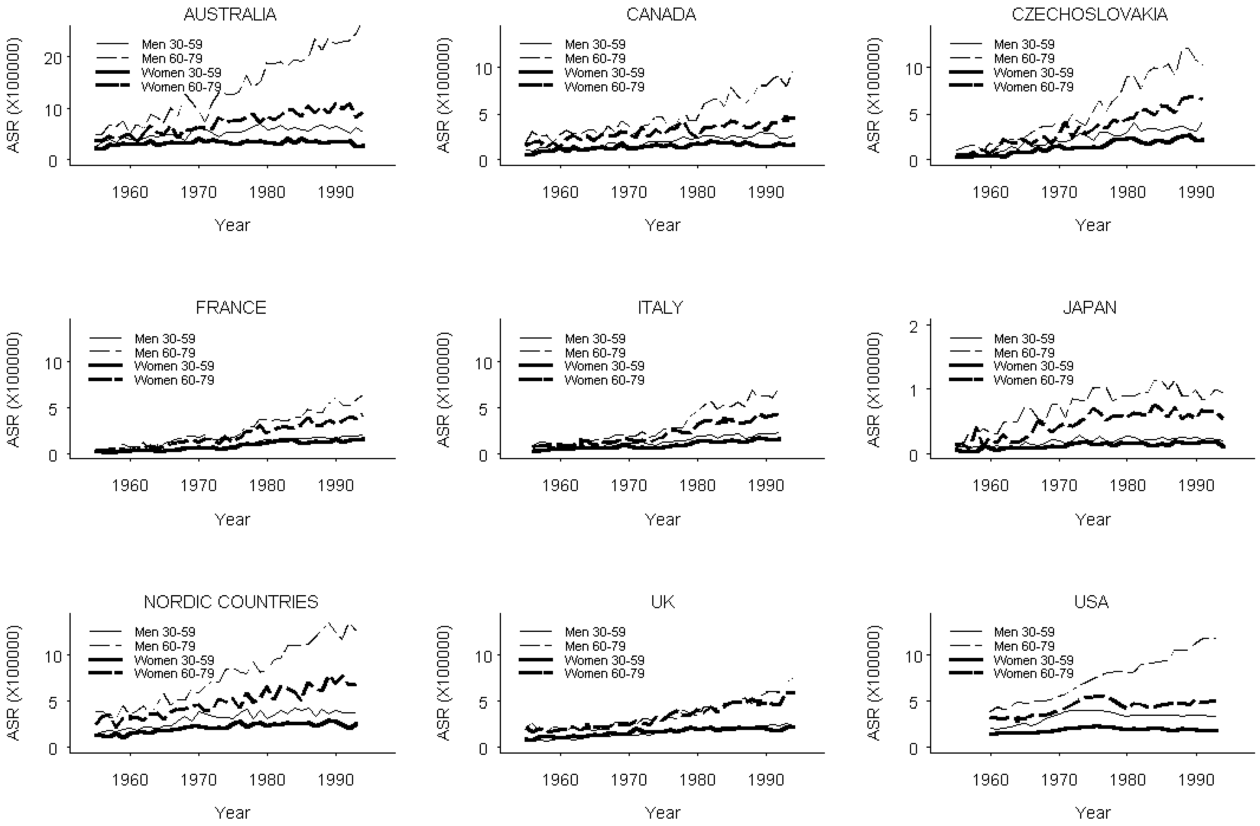

**Figure 1** Age-adjusted mortality rates for young (30–59) and old (60–79) men and women (world standard population)

(relative to Whites) and slightly the magnitude of trends but not the trend patterns.

Variations in mortality rates can reflect the combined effects of birth cohort, period and age; the most effective and reliable way to examine their respective influences is via age–period–cohort (APC) models. Such models yield a meaningful estimate of the ‘drift’ – a measure of the linear increase/decrease of mortality rates over time. Drift is the sum of both linear period and birth cohort effects. It is impossible to separate unequivocally these two effects because age, period and cohort of birth are not independent (Holford, 1983). As a consequence, estimates of mortality rates by cohort of birth (combination of drift and non-linear effects) vary according to the attribution of drift to period or to birth cohort. We, therefore, chose to perform a sensitivity analysis by graphing the estimates after attributing: (1) the entire drift to a period effect, (2) the entire drift to a birth cohort effect and (3) half the drift to a period effect and half to a birth cohort effect. Studies published almost two decades ago (Lee et al, 1979; Venzon and Moolgavkar, 1984) suggested that the rise in CMM mortality was following a birth cohort pattern. Hence, the scenario where all the drift is attributed to a period effect is improbable. In drawing conclusions from this analysis it should be noted that the most recent generations contain few deaths and consequently the corresponding rate estimates have relatively large standard errors.

For the APC models, data were grouped in 5-year periods. Hence birth cohorts are in 10-year groups with a 5-year overlap between adjacent groups. In the graphs, birth cohorts are identified by their middle year. We restricted the age range to 30–84 years in order to avoid age groups with small numbers of deaths. We followed the implementation of APC models described by Holford (1992) using the statistical package S-plus 4.5 (Mathsoft). Further details of the APC analysis are available on request.

### RESULTS

### Trends in the age-standardized rates

Age-standardized CMM mortality rates for the 30–59 and 60–79 age groups are shown in Figure 1. Australia and Japan are drawn to different scales because of the different magnitude of the mortality rates observed in these countries.

In the age group 30–59, in 1960, CMM mortality rates were low in all countries, and equivalent in men and women. In France, Italy and Czechoslovakia, there were fewer than 0.5 deaths due to CMM per 100 000 annually, while in Australia and the USA, there were 2 deaths per 100 000 – other countries having intermediate values. Over the period 1960–1990, in Australia, CMM mortality in the 30–59 age group increased annually by an average 2% in men, while it remained almost stable in women. A similar pattern was observed in the USA, with an average annual increase of 2% in men and of 1% in women. The highest rates of increase in CMM mortality rates were found in France, Italy and Czechoslovakia, with death rates increasing annually by 9–16% without any significant sex difference.

In the age group 60–79, increases in CMM mortality rates were greater than in the 30–59 age group, and the most dramatic

 *© 2000 Cancer Research Campaign*

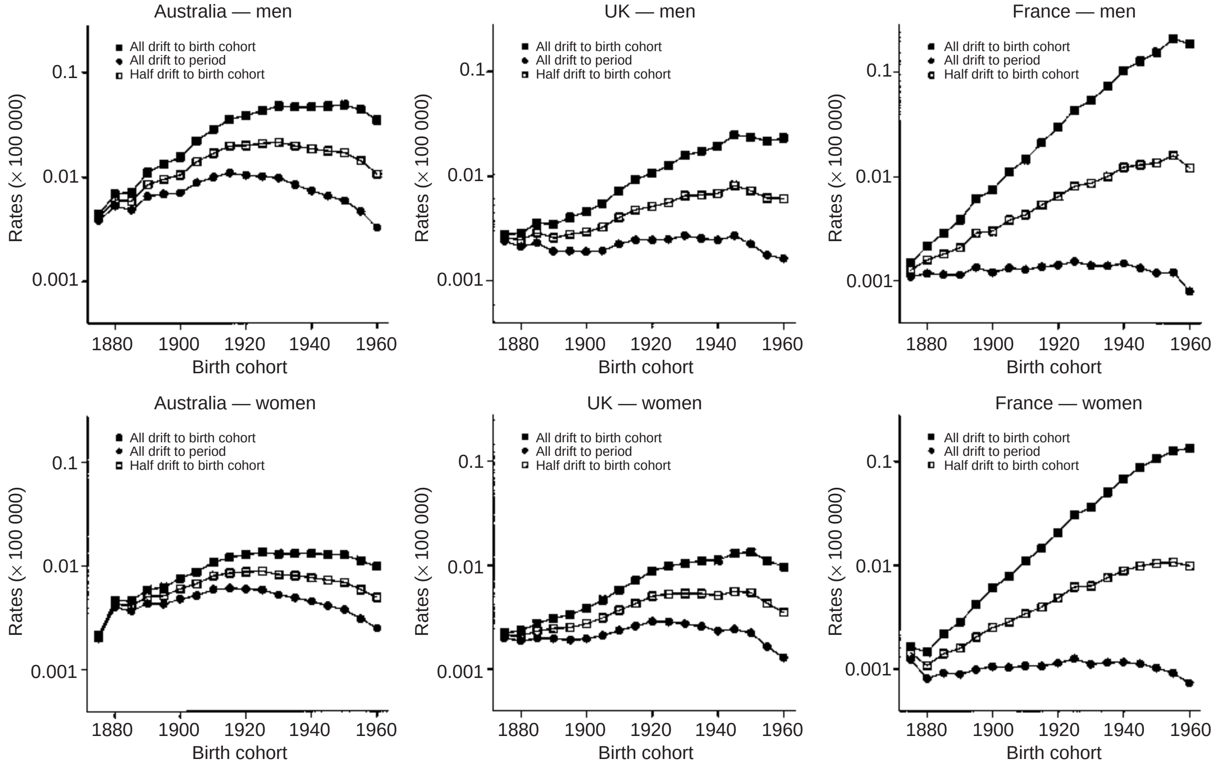

**Figure 2** Trends in mortality rates from the age–period–cohort model for men and women in three selected countries increases were observed among men. The annual percentage increase in rates for men and women 60–79 years old was, respectively, 12% and 4% in Australia, 7% and 2% in the USA, and 9% and 4% in the Nordic countries. We found a less marked difference between men and women in the CMM mortality increase in Canada (6% for men and 4% for women), Japan (6% and 4%) and the UK (7% and 5%). In France, Italy and Czechoslovakia, CMM mortality rates were low in the 1960s, but afterwards, average annual increases were 24% and 27% in French men and women, 15% and 13% in Italian men and women, and 17% and 33% in Czechoslovakian men and women. Because CMM mortality increased more in older than in younger people, those who died from CMM in 1994 were 10 years older on average than those who died in 1960.

### Age–period–cohort models

The age–period–cohort models yielded drift terms (measures of the average trend over time) that were all positive and in agreement with the annual percentage increases described above. CMM mortality rose linearly during the period of observation in every population. In every country but Japan, the increase was greater (higher drift) for men than for women. The largest sex differences in drift were observed in Australia, Canada and the USA. Consistent with the analysis of the age-standardized rates, the highest values for drift were found in France, Italy and Czechoslovakia.

Trends in mortality rates by birth cohort have been derived from the APC model, and are shown in Figure 2 for three selected countries (Australia, UK and France). These countries were chosen because they represented the three CMM mortality patterns that we observed after APC modelling (see below). Each graph contains three plots that described different scenarios: in the upper plot, all the drift is attributed to a birth cohort effect, in the lower plot all the drift is attributed to a period effect. The rates displayed in Figure 2 simply reflect the trend in rates by birth cohort and are therefore not comparable with actual rates observed in populations. If in each scenario rates are decreasing, we can conclude that there is a real decrease. The trends in CMM mortality rates illustrated by these models describe three patterns:

1. increasing rates until generations born around 1930–1935 (for Australia increasing rates ceased earlier), followed by decreasing rates in more recent birth cohorts (in Australia, the Nordic countries and the USA)
2. increasing rates until generations born during World War II, followed by flattening or slightly decreasing rates in more recent birth cohorts (UK, Canada)
3. a steep, almost linear, increase with no major change in this trend (France, Czechoslovakia, and Italy).

CMM mortality rates in Japan showed a pattern similar to that described in the third group but the rates of increase were much smaller.

### DISCUSSION

We have succeeded in identifying meaningful patterns in the global CMM epidemic using data only from populations with good quality death registration and relatively lengthy time series. Our analysis suggests that populations are at different places on the

© 2000 Cancer Research Campaign

CMM mortality epidemic curve and that they fall into three distinct groups. In some the mortality trend is still strongly waxing, in others it is beginning to wane, and in a few, it is definitely diminishing.

The global increase in CMM mortality among fair-skinned populations has long been regarded as a consequence of increasing sun exposure to people sensitive to the harmful effects of sunlight. Recent decreases in CMM mortality that have been observed in some populations have been attributed to the success of skin cancer prevention programmes. This is an attractive assertion that is difficult either to prove or disprove, though there is some evidence from Scotland that interventions can achieve changes associated with reduced mortality (MacKie and Hole, 1992). Most of these programmes have combined messages of solar protection with those of early detection, and there is some epidemiological evidence that reducing sun exposure in childhood reduces CMM risk (Autier and Doré, 1998), but there is little evidence that this is true in adults (English and Milne, 1999). If prevention programmes are effective in reducing CMM mortality, early effects should be noticeable in the younger age groups rather than in the older ones.

On face value, the trend in age at death is encouraging – in every population the median age at death has increased between 1960 and the early 1990s, people dying from CMM in 1994 being 10 years older on average than those who died in 1960. However, this is mostly due to the greater increase in CMM mortality rates among older people compared with younger people. Three mechanisms may have contributed towards this age contrast in CMM mortality:

1. It is probable that changes towards safer sun exposure habits in recent decades have been greater in the young. As age is a marker of lifetime sun exposure, CMM susceptible people who were 60–79 years old in 1994 experienced a lifetime sun exposure higher than similar people of the same age in 1960. Given the importance of sun exposure during childhood to CMM in adult life (Autier and Doré, 1998), the continuously increasing CMM mortality observed among older people could be the consequence of heavy sun exposure when they were young
2. The decreasing thickness of CMM, reported in several countries, has been more pronounced in younger people (Van der Spek-Keijser et al, 1997; Bucchi et al, 1992)
3. The improvement in survival from CMM does not seem to be entirely attributable to earlier stage at diagnosis or to better medical management (Masbach et al, 1994). It is possible that the disease of ‘CMM’ has decreased in aggressiveness. Recent epidemiological data (Autier and Doré, 1998; Burton and Armstrong, 1994) suggest that changes in CMM behaviour could result from variations in the amounts of sun exposure experienced during different periods of life.

The increase in mortality has often been greater for men than for women. CMM is known to have a worse prognosis in men (MacKie et al, 1992; Thörn et al., 1994; Smith et al., 1998). Some data also show a sex difference in effectiveness of CMM prevention campaigns. For example, in Scotland, campaigns resulted in higher proportions of thin CMMs in women than in men (MacKie and Hole, 1992). The effects on mortality induced by a sex difference in CMM awareness would be amplified by the sex difference in anatomic distribution. In men, CMM arises more often on the back and on the head and neck where CMMs carry a poorer prognosis than those detected at other sites. In women, the increase in CMM incidence has been greater on the legs, where it has a better prognosis and is easier to detect by self-examination.

### Is the end of the increasing trend in sight?

The three patterns in CMM mortality that we have described correlate with time differences in the initiation of large-scale skin cancer prevention programmes encouraging sun protection and surveillance of pigmented skin lesions. The medical literature indicates that these did not start at the same time in all countries: in some Australian states, prevention programmes started in the 1960s (Giles et al, 1996) and then national programmes (‘Slip, Slop, Slap’ and ‘Sun Smart’) followed in the 1980s. In the USA, Canada, UK and Sweden, national programmes started in the 1980s or early 1990s (Robinson et al, 1997; Rivers and Gallagher, 1995; Melia, 1995; Möller et al., 1997). In France, Italy and Czechoslovakia, however, we found no report on organized long-term national programmes. Other reports suggest that the latter situation pertains to most Southern and Central European countries (La Vecchia et al, 1999; Bleyen et al, 1999).

In the first group of populations the epidemic of CMM mortality has peaked and, unless population behaviour changes to an increase in risky exposures, it is likely that further declines in mortality will be experienced over the coming years. In the second group the signs are encouraging but further gains may be enhanced by sustained and increasingly resourced prevention campaigns. The third group of largely central and southern European countries are still experiencing an unchecked increase in CMM deaths, and, in the absence of effective skin cancer prevention programmes, their CMM mortality can be expected to rise unabated.

### REFERENCES

Armstrong BK and Kricker A (1994) Cutaneous melanoma. *Cancer Surv* **19**: 219–240

Autier P and Doré JF (1998) Influence of sun exposures during childhood and during adulthood on melanoma risk. *Int J Cancer* **77**: 533–537

Balzi D, Carli P and Geddes M (1997) Malignant melanoma in Europe: changes in mortality rates (1970–1990) in European Community countries. *Cancer Causes Control* **8**: 85–92

Bleyen L, De Bacquer D, Myny K et al (1999) Trends in mortality from cutaneous malignant melanoma in Belgium. *Int J Epidemiol* **28**: 40–45

Bucchi L, Serafini M and Lanzanova G (1992) Breslow thickness of cutaneous malignant melanoma in Ravenna (northern Italy) 1981–1990. *Tumori* **78**: 94–97

Burton R and Armstrong B (1994) Recent incidence trends imply a non-metastasising form of invasive melanoma. *Melanoma Res* **4**: 107–113

English DR and Milne E (1999) Favorable trends in melanoma incidence: can we claim credit? *Cancer Causes Control* **10**: 403–405

Giles GG, Armstrong BK, Burton RC et al (1996) Has mortality from melanoma stopped rising in Australia? Analysis of trends between 1931 and 1994. *Br Med J* **312**: 1121–1125

Holford TR (1983) The estimation of age, period and cohort effects for vital rates. *Biometrics* **39**: 311–324

Holford TR (1992) Analysing the temporal effects of age, period and cohort. *Stat Methods Med Res* **1**: 317–337

LaVecchia C, Lucchini F, Negri E et al (1999) Recent trends in worldwide mortality from cutaneous melanoma in youth and middle age. *Int J Cancer* **81**: 62–66

Lee JAH, Petersen GR, Stevens RG et al (1979) The influence of age, year of birth, and date on mortality from malignant melanoma in the populations of England and Wales, Canada, and the white populations of the United States. *Am J Epidemiol* **110**: 734–739

MacKie RM and Hole H (1992) Audit of public education campaign to encourage earlier detection of malignant melanoma. *Br Med J* **304**: 1012–1015

MacKie RM, Hunter JAA, Aitchison TC et al (1992) Cutaneous malignant melanoma in Scotland 1979–89. *Lancet* **339**: 971–975

*© 2000 Cancer Research Campaign*

MacKie RM, Hole D, Hunter JAA et al (1997) Cutaneous malignant melanoma in Scotland: incidence, survival, and mortality, 1979–1994. *Br Med J* **315**: 1117–1121

Masbach A, Westerdahl J, Ingvar C et al (1994) Cutaneous malignant melanoma in South Sweden 1965, 1975, and 1985. *Cancer* **73**: 1625–1630

MathSoft, Inc (1998) S-plus user's manual. Seattle

Melia J (1995) Early detection of cutaneous malignant melanoma in Britain. *Int J Epidemiol* **24** (Suppl 1): S39–44

Möller TR, Hult C, Isacsson Å and Lindholm LH (1997) Multimedia techniques in the primary and secondary prevention of malignant melanoma. In *Skin Cancer and UV Radiation*, Altmeyer P, Hoffmann K, Stücker M (eds), pp. 930–941. Springer-Verlag: Berlin

Rivers JK and Gallagher RP (1995) Public education projects in skin cancer. Experience of the Canadian Dermatology Association. *Cancer* **75** (2 Suppl): 661–666

Robinson JK, Rigel DS and Amonette RA (1997) Trends in sun exposure knowledge, attitudes, and behaviors: 1986 to 1996. *J Am Acad Dermatol* **37**: 179–186

Roush GC, McKay L and Holford TR (1992) A reversal in long-term increase in deaths attributable to malignant melanoma. *Cancer* **69**: 1714–1720

Scotto J, Pitcher H and Lee JAH (1991) Indications of future decreasing trends in skin melanoma mortality among Whites in the United States. *Int J Cancer* **49**: 490–497

Smith JAE, Whatley PM and Redburn JC (1998) Improving survival of melanoma patients in Europe since 1978. *Eur J Cancer* **34**: 2197–2203

Swerdlow AJ (1990) International trends in cutaneous melanoma. *Annals of the New York Academy of Sciences* **609**: 235–251

Thörn M, Pontén F, Bergström R, Sparén P and Adami HO (1994) Clinical and histopathologic predictors of survival in patients with malignant melanoma: a population-based study in Sweden. *J Natl Cancer Inst* **86**: 761–769

Van der Spek-Keijser LMT, Van der Rhee HJ, Toth G et al (1997) Site, histological type and thickness of primary cutaneous malignant melanoma in western Netherlands since 1980. *Br J Dermatol* **136**: 565–571

Venzon DJ and Moolgavkar SH (1984) Cohort analysis of malignant melanoma in five countries. *Am J Epidemiol* **119**: 62–70

*© 2000 Cancer Research Campaign*
